# Intermittent Claudication

**Published:** 1908-12-12

**Authors:** 


### Medicine.

### INTERMITTENT CLAUDICATION.

Intermittent claudication, or limping, also known as angina cruris, is a condition which has recently been attracting considerable attention as the result of publication of cases by Dr. J. Ramsay Hunt of New York, Dr. F. Parkes Weber, Mr. A. Pearce Gould in his Lettsomian lectures, Dr. Byrom Bramwell in his Clinical Studies, and so forth. The disease was originally described by Charcot in 1858, and Professor Erb published a monograph upon it in 1898. Though not common, the condition is so very striking that it is immediately recognised. Dr. Byrom Bramwell's case* was very typical, as to both symptoms and pathology; it may serve as a clinical description of the disease.

The patient was a married man, aged forty-six. He denied venereal infection, and there was no clinical evidence of it. Until eighteen months before he first came under observation he had enjoyed excellent health, having been a steward on board a liner for five years. Previous to this he had been in the army for twenty years, during which time he was an athlete, a runner, and a swimmer. There had been no excess as regards alcohol or smoking, and there had been no undue privation or exposure. His wife was healthy, there were three healthy children, and there had been no miscarriage.

The patient was perfectly well until September, 1905. He then had an attack, diagnosed as influenza. In October, 1905, he began to suffer from pains in the right leg shooting from the hip to the ankle. Soon afterwards he noticed that walking for any distance caused pain, stiffness, and cramp in the right leg, and that if he persisted and tried to walk off the stiffness, the leg became extremely rigid, and the tendons stood out as if the parts were being forcibly drawn asunder. So long as he was at rest there was, he said, no pain, weakness, or stiffness in the leg, but walking a short distance—300 yards—would bring on these symptoms, and make him limp. After resting for a short time—half an hour or so—the pain, stiffness, and weakness passed off. He stated that when the pain and weakness came on in the leg, the veins used to look as if they were full of blood; the leg, more especially the foot, became dark in colour, and little red spots were apt to develop on the surface of the skin.

The above is a very striking and characteristic account of the affection known as intermittent claudication, the essential clinical features of which are:—

(1) Absence of symptoms whilst the affected limb is at rest.

(2) The development of symptoms—acute and painful limping—after exertion.

(3) Subsidence of the symptoms again on resting.

(4) Absence of pulsation in the pedal arteries.

The significance of the last point will be more apparent when the pathology of the condition is referred to presently.

* *Clinical Studies.* Edinburgh. 1908. P. 317.

In typical cases the patient feels quite well until he puts the muscles of the affected part into prolonged action. In some cases, however, more especially in the advanced stages of the disease, weakness, and it may be even muscular atrophy, paræsthesiæ and vaso-motor alterations are persistently present—that is to say, they are present independently of exertion. In the advanced stage of the disease dry gangrene may be developed, and severe continuous pain in the affected part results.

After walking a longer or a shorter distance, which varies in different cases, motor and sensory symptoms, and vaso-motor alterations, due it is believed to insufficient arterial blood supply to the affected part, are developed. The more important of these symptoms are:—weakness, stiffness, heaviness, coldness, numbness, a prickling feeling, pain, cramps, and rigidity in the affected limb, or limbs. In consequence of the weakness, stiffness, pain and cramp, the patient limps; and as the condition is not present during rest, but only after exertion, it comes on intermittently. The affected limb may present marked evidence of derangement of the circulation. The foot may become red and swollen, or the toes become white and dead. During the paroxysm the affected limb may be colder to the touch than is its fellow.

The development of the symptoms—not unlike string-halt in horses—is believed to be due to defective blood supply in the affected part. There may be enough blood for the needs of the part when it is at rest, but increased requirements of exertion cannot be met. So long as the patient is at rest there are no symptoms, at any rate in the earlier stages of the malady. During rest the feeble arterial blood supply is sufficient for the tissue requirements; but during active exertion the increased demand which the muscular contractions entail cannot be satisfied, with the result that symptoms arise from the acute relative ischæmia.

In most cases of intermittent claudication no pulsation can be felt in the pedal arteries. This is an important diagnostic point; in all cases in which a patient complains of intermittent leg pains and limping after exertion, the condition of the arterial circulation should be carefully examined. The absence of pulsation in the pedal arteries may be due to anything which obstructs the vascular channel. In most cases the obstruction is due to organic narrowing of the lumen by obliterative endarteritis; but in addition to organic changes there is often a functional vaso-motor spasm also. When the latter factor is at all prominent, the features of the case may be not unlike those of Raynaud's disease. Thus, in Dr. Bramwell's patient, local syncope and local asphyxia developed some little time after the intermittent claudication was first obvious. Small red spots about the size of a threepenny piece began to appear on the affected leg. These spots were very painful to the touch. They were only slightly, if at all, raised above the surface of the skin. They developed in successive crops, on the outer side of the thigh from the hip to the knee, and on the inner side of the leg from the knee to the ankle. The development of the spots was preceded by a feeling of pain and stinging in the skin. Each crop of spots would last two or three days, and then disappear; before they faded, the spots became darker in colour. A new crop of spots would then develop in the course of three or four days, and this process continued for several months.

The disease may go on for many years. The ultimate result, as might be expected from the state of the arteries, is dry gangrene of the toes and feet. This occurred in Dr. Bramwell's patient, first in one leg, and after an interval, in the other. The treatment of the disease is clearly palliative only. A clear conception of the pathology of the affection will lead to appropriate treatment at different stages —strict control of the amount of exercise, though not complete rest, except in late stages; massage; electrical and allied treatment when vaso-motor spasm is a marked feature; iodides and arsenic; prophylaxis against injury to the part, or anything that might cause gangrene to set in; appropriate surgical measures when gangrene has developed.

